# miR-543 promotes colorectal cancer proliferation and metastasis by targeting *KLF4*

**DOI:** 10.18632/oncotarget.19495

**Published:** 2017-07-22

**Authors:** Fangbing Zhai, Chunhong Cao, Liang Zhang, Jianhua Zhang

**Affiliations:** ^1^ Department of Radiology, The Second Affiliated Hospital of Dalian Medical University, Dalian 116027, Liaoning, China; ^2^ Department of Interventional Therapy, The Second Affiliated Hospital of Dalian Medical University, Dalian 116027, Liaoning, China; ^3^ Department of Nursing, The Second Affiliated Hospital of Dalian Medical University, Dalian 116027, Liaoning, China

**Keywords:** miR-543, KLF4, CRC, proliferation, metastasis

## Abstract

Till now, miR-543 expression has been demonstrated to be involved in the development of some cancers. However, reports about its expression and mechanism in colorectal cancer (CRC) were conflicting [1, 2]. Here, we investigated clinical implications of miR-543 and mechanisms underlying miR-543-mediated CRC development. In this study, real-time quantitative PCR (qRT-PCR) validated miR-543 was highly expressed in CRC samples and cell lines. MiR-543 was closely associated with tumor size, TNM stage and metastasis. In addition, survival analysis showed that high miR-543 expression was obviously correlated with poor overall survival and disease-free survival. Mechanically, downregulation of miR-543 by miR-543 inhibitor obviously repressed cell proliferation, promoted apoptosis, affected migration and invasion. Moreover, luciferase reporter analysis identified that Krüppel-like Factor-4 (*KLF4*) was a direct target of miR-543, and there was an obvious inverse correlation between miR-543 and *KLF4* expression in CRC tissues. Furthermore, *KLF4* down-regulation favors miR-543-induced oncogenic effect on cell proliferation, apoptosis, migration, and invasion. In conclusion, this study indicated that miR-543 facilitates colorectal cancer proliferation and metastasis by targeting *KLF4*, and miR-543 may serve as a promising target for the treatment of CRC patients.

## INTRODUCTION

MicroRNAs (miRNAs) are a class of small (19~24 nucleotides), non-coding RNA molecules that play important role in the pathogenesis of colorectal cancers [3–7]. Altered expression of miRNAs in colorectal cancer was first described by Michael and co-workers in 2003 [8]. Subsequently, association of colorectal cancer pathogenesis and hundreds of miRNAs were demonstrated [3, 9]. These miRNAs play crucial roles in various cellular processes such as cell proliferation, cell cycle, apoptosis, maintenance of stemness, epithelial to mesenchymal transition (EMT), differentiation, angiogenesis and metastasis of colorectal cancer cells [10–12]. They modulate carcinogenesis of colorectal cancer by activating oncogenes and inactivating tumor suppressor genes, or DNA repair genes [3, 9].

It has been reported that the expression of miR-543 is obviously downregulated in breast cancer [13] and endometrial cancer [14], however, the expression of miR-543 is obviously up-regulated in hepatocellular carcinoma [15] and prostate cancer [16]. Sun J et al reported that miR-543 was constitutively increased in CRC samples and its over-expression enhanced cancer cell migration and invasion. Furthermore, they verified PTEN as a direct target of miR-543 by a dual-luciferase reporter assay. Thus, miR-543 serves as a tumor promoter [1]. However, Fan C et al reported that miR-543 affects cell proliferation and metastasis of CRC by targeting the expression of KRAS, MTA1 and HMGA2. Thus, miR-543 serves as a suppressor in CRC growth and metastasis [2]. Obviously, these results had conflicts. Thus it is essential to demonstrate the real role of miR-543 in CRC by our experiments.

In the present study, we aims to examine the expression profiling as well as clinicopathological and prognostic implications of miR-543 in a large series of patients with colorectal cancer. *In vitro* miR-543 mediated cellular effects and target proteins expressions in colon cancer were also examined.

## RESULTS

### Expression of miR-543 in CRC tissues and cell lines

**Figure 1:**
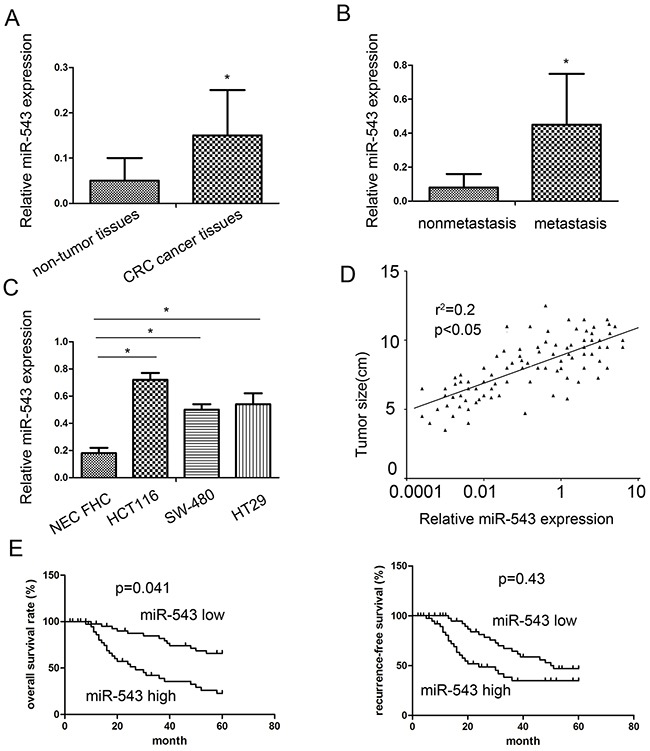
miR-543 is overexpressed in primary CRC tumor tissues and cell lines and correlates with patient survival **(A)** The miR-543 expression in 92 cases of primary CRC tissues compared with 20 cases of adjacent nontumorous tissues analyzed by RT-qPCR. **(B)** The miR-543 expression in primary CRC tissues with lymph node metastasis compared with those without lymph node metastasis. **(C)** miR-543 expression in CRC cell lines. **(D)** miR-543 expression was positively associated with tumor size by Spearman's correlation test (*n* = 92). **(E)** The correlation between miR-543 expression and CRC patient survival. * *p* < 0.05; the data represent the mean ± standard deviation (SD) from triplicate measurements.

In this study, we explored the expression of miR-543 using qRT-PCR in CRC cancer samples and adjacent non-tumor samples. We found that miR-543 expression in cancer tissues was obviously higher compared with that in adjacent non-tumor tissues (Figure 1A). miR-543 expression in patients with metastasis was obviously higher compared with those without metastasis (Figure 1B). Consistent with results in tissues, miR-543 was also highly expressed in CRC cell line HCT116 and SW-480 compared with human intestinal epithelial cell lines FHC (Figure 1C). To further explore the clinic role of miR-543 expression in CRC tissues, 92 CRC patients were separated into two groups for survival analysis according to miR-543 expression involving the low miR-543 expression group and the high miR-543 expression group. Our clinicopathologic data validated that the miR-543 overexpression was associated with tumor size, TNM stage and lymph node metastasis (Table 1). Furthermore, spearman correlation analysis revealed that miR-543 expression was positively correlated with tumor size (Figure 1D).

**Table 1:** Association between miR-543 expression and clinicopathologic parameters in CRC patients

Characteristics	Number	Median	*p* Value
Age (years)			
≥60	52	0.625	0.113
<60	40	0.350	
Gender			
Male	54	0.599	0.226
Female	38	0.391	
Tumor size (cm)			
<5	44	0.324	0.033*
≥5	48	0.676	
Differentiation status			
Well/moderate	45	0.613	0.089
Poor	47	0.399	
TNM stage			
I + II	32	0.354	0.023*
III + IV	60	0.588	
Lymph node metastasis			
Negative	36	0.301	0.036 *
Positive	56	0.632	

* *p* < 0.05 was considered significant.

### miR-543 is correlated with poor prognosis of CRC patients

Kaplan–Meier survival analysis and log-rank test were applied to assess the prognostic implications of miR-543 expression in CRC patients. We found that patients with high miR-543 expression had significantly shorter overall survival (OS) and disease free survival (DFS) than those with low miR-543 expression (Figure 1E), suggesting that miR-543 may act as a promising and useful candidate for the prognosis of CRC patients.

### miR-543 regulates proliferation and apoptosis

**Figure 2:**
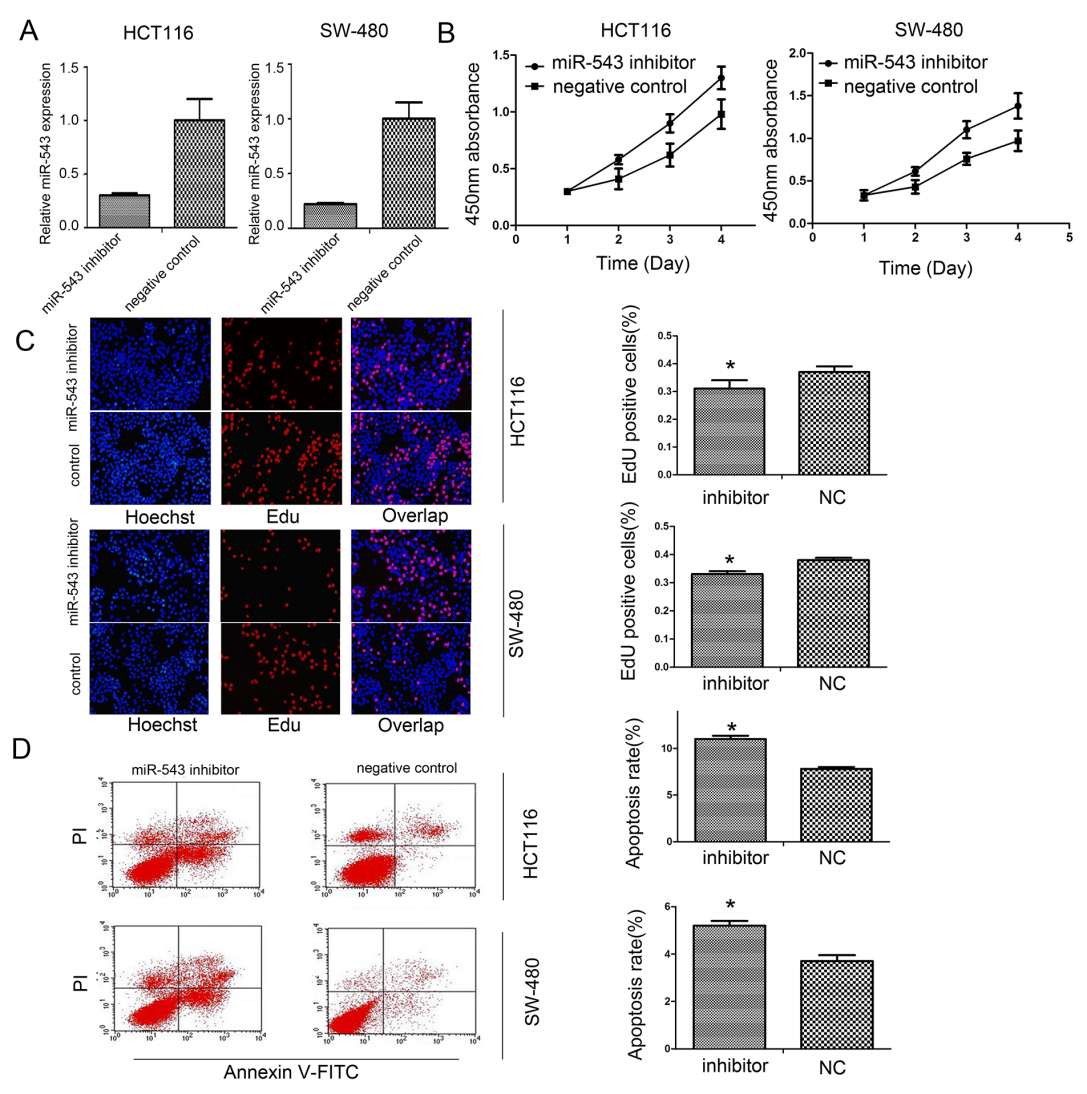
miR-543 promotes cell proliferation and reduced apoptosis of CRC cells **(A)** Analyses of miR-543 expression after transfection in HCT116 and SW-480 cells by real-time PCR. **(B, C)** Influence of miR-543 downregulation on cell proliferation of HCT116 and SW-480 cells by CCK-8 (B) and EdU assays. **(C, D)** Influence of miR-543 knockdown on cell apoptosis. * *p*<0.05, Scale bar = 100 μm for (C); the data represent the mean ± SD from triplicate measurements.

As shown in Figure 1C, HCT116 and SW-480 cells showed a relatively higher miR-543 expression. So, we conducted miR-543 inhibitor transfection in both cell lines. To demonstrate transfection efficiency, qRT-PCR was used to detect the expression of miR-543 at post-transfection 48 h. Our results showed that relative miR-543 expression was obviously lower in HCT116 and SW-480 cells transfected with miR-543 inhibitor compared with that in negative control (NC) groups (Figure 2A).

To investigate whether miR-543 was able to affect CRC cells proliferation, CCK-8 and EdU assays were applied to evaluate cell growth status. These findings showed that downregulation of miR-543 could decrease growth of HCT116 and SW-480 cells (Figure 2B, 2C). In addition, miR-543 inhibitor also induced the apoptosis of HCT116 and SW-480 cells compared with the negative control (Figure 2D).

### Reduction of miR-543 inhibited CRC migration and invasion

**Figure 3:**
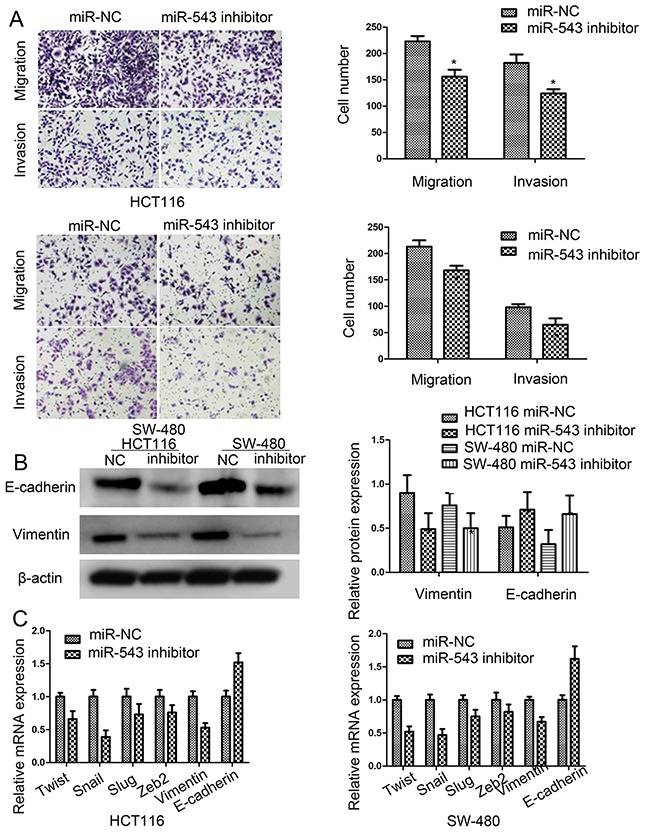
miR-543 promotes cell migration, invasion and mesenchymal-epithelial transformation (EMT) of CRC cells **(A)** Effect of miR-543 knockdown on cell migration and invasion ability in HCT116 and SW-480 cells, Scale bar = 50 μm. **(B)** E-cadherin and vimentin expression in HCT116 and SW-480 cells by western blot. **(C)** Epithelial-to-mesenchymal transition (EMT)-associated genes expression in HCT116 and SW-480 cells by RT-qPCR. * *p* < 0.05; the data represent the mean ± SD from triplicate measurements.

In this study, transwell assays were used to assess the effects of miR-543 on cell migration and invasion. When the expression of miR-543 was blocked, HCT116 and SW-480 cell migration and invasion capability were significantly reduced (Figure 3A).

### miR-543 knockdown repressed the epithelial-mesenchymal transition (EMT)

To explore whether miR-543 is implicated in EMT progression of CRC, we detected the expression of varieties of EMT biomarkers. Our results showed that downregulation of miR-543 increased the expression of E-cadherin, and decreased the expresison of vimentin in CRC cells (Figure 3B). Overall, most of EMT-related genes were down-regulated by miR-543 knockdown (Figure 3C). All in all, these results validated that knockdown of miR-543 could inhibit the EMT processes of CRC cells.

### miR-543 downregulation represses *in vivo* CRC growth and lung metastasis

**Figure 4:**
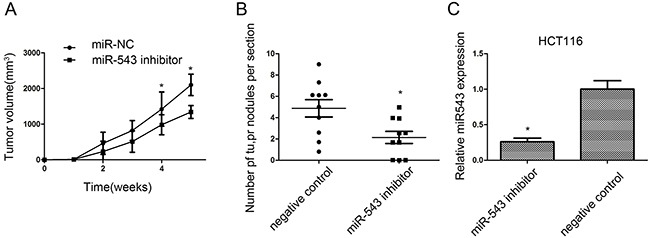
miR-543 promotes CRC cell growth and metastasis *in vivo* **(A)** Tumor volumes were measured on the indicated days. **(B)** The number of lung metastasis of indicated SCID mice groups. **(C)** RT-qPCR analysis of miR-543 expression in implanted tumors. * *p* < 0.05; the data represent the mean ± SD from triplicate measurements.

In the present study, the growth of HCT116 xenograft was significantly affected by knockdown of miR-543 (Figure 4A). The lung metastases of HCT116 xenograft were also significantly repressed by downregulation of miR-543, and the number of lung metastatic nodules was also reduced when compared with the negative controls (Figure 4B). At the same time, RT-qPCR test revealed that relative miR-543 expression in the tumor xenograft was lower in HCT116 cells with lentivirus miArrest™ miR-543 inhibitor compared with that in negative control (Figure 4C).

### miR-543 directly down-regulates KLF4 expression

**Figure 5:**
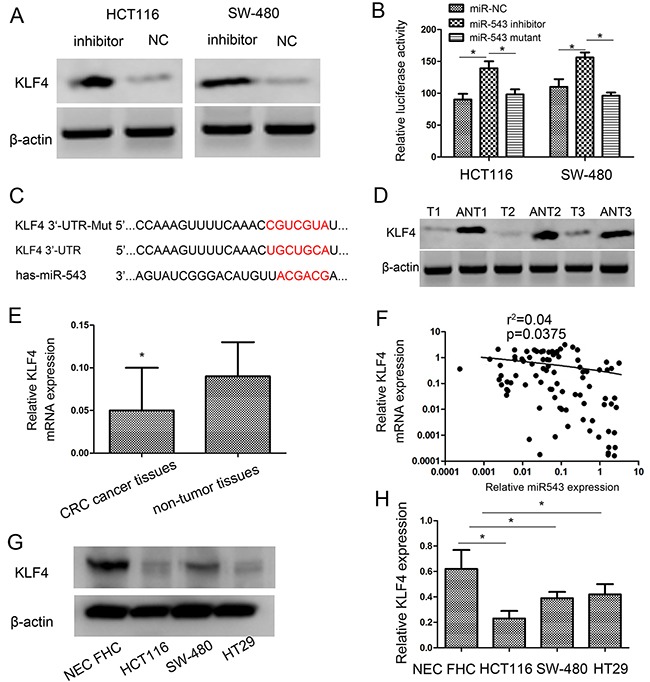
*KLF4* is a direct target of miR-543 and *KLF4* expression is inversely correlated with miR-543 expression in CRC tissues **(A)** Western blot analysis of KLF4 protein expression after transfection in HCT116 and SW-480 cells. **(B)** Luciferase activities of wild-type and the mutant pmirGLO-*KLF4*-3′-UTR reporter in HCT116 and SW-480 cells. **(C)** The predicted miR-543 binding site on the *KLF4* mRNA 3′-UTR and the corresponding mutations in 3′-UTR of *KLF4*. **(D, E)** KLF4 expression on protein level (D) and mRNA level (E) was determined in CRC tissues (T) and adjacent nontumorous tissues (ANT). **(F)** Spearman's correlation analysis was performed to detect the association between the expression level of miR-543 and *KLF4* in GC tissues. **(G, H)** KLF4 expression on protein level (G) and mRNA level (H) was determined in three CRC cell lines (HCT116, SW-480 and HT29) and nontumorous mucosa. Error bars represent mean ± SD from three independent experiments. * *p* < 0.05.

To explore the molecular mechanisms underlying miR-543-induced biological processes, 4 cancer-associated (*DICER1*, *CPEB3*, *FOXP1* and *KLF4*) were predicted by three common databases (Pictar, Miranda, and Targetscan), and then genes were selected as candidates to be explored. As illustrated in Figure 5A, immunoblot analysis revealed that the expression of KLF4 protein was obviously increased in HCT116 and SW-480 cells with miR-543 inhibitor than those with the negative control. Previous work also demonstrated that *KLF4* was a proliferation- and metastasis-associated gene in CRC development. According to these results, we assumed that *KLF4* should be a target of miR-543 in CRC.

Consistent with the Western blot analysis, when we transfected miR-543 inhibitor into cells, the activity of a luciferase reporter gene with *KLF4* 3′-UTR was obviously increased by 36.02 ± 1.5% and 28.51 ± 1.6% respectively in HCT116 and SW-480 cells compared with negative control (Figure 5B). To demonstrate that *KLF4* acts as a direct target of miR-543, *KLF4* 3′-UTR and its mutant were constructed into pmirGLO luciferase reporter plasmid. Downregulation of miR-543 increased luciferase activity in HCT116 cells with the wild-type 3′-UTR of *KLF4* but not in HCT116 cells with mutant 3′-UTR (Figure 5B). Similar findigns were found in SW-480 cells (Figure 5B). These findings indicated that *KLF4* was a direct target of miR-543 in CRC, and the miR-543 binding site existed in the position of 541-547 of 3′-UTR of *KLF4* (Figure 5C).

### miR-543 and KLF4 are clinically correlated in CRC tissues and cells

To figure out the clinical correlation of miR-543 and *KLF4* in CRC, the expression of *KLF4* mRNA and protein in cancer and normal tissues were analyzed usign western blot and qRT-PCR analysis. Our findings validated that *KLF4* expression was decreased in CRC tissues (Figure 5D, 5E). Moreover, there was an inverse correlation between miR-543 and *KLF4* expression (*r*^2^= 0.04, p=0.0375; Figure 5F). Additionally, the expression of *KLF4* in CRC cell lines was also detected. Western blot and qRT-PCR results showed that *KLF4* expressed at a lower level in CRC cell lines compared with nontumorous mucosa (Figure 5G, 5H). These findings further suggested that *KLF4* expression was negatively regulated by miR-543 in CRC. These results indicated that miR-543 had an inhibitory effect on *KLF4* in CRC.

### Down-regulation of KLF4 favors oncogenic effects of miR-543

**Figure 6:**
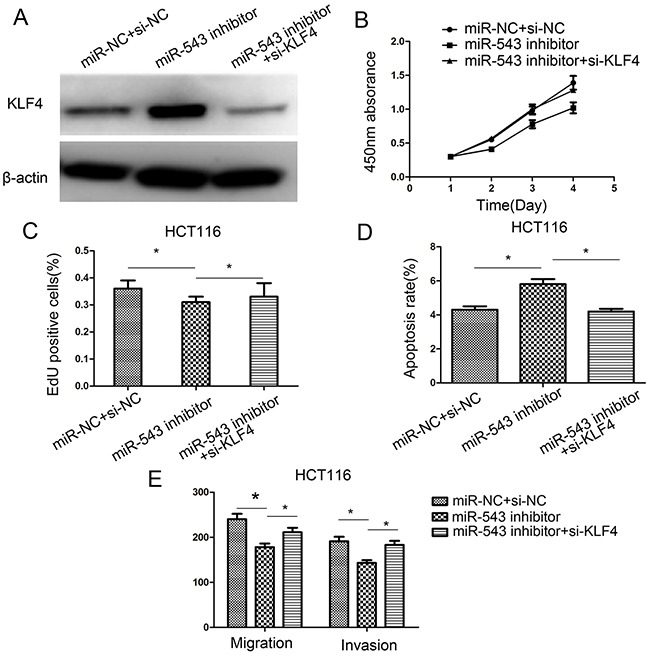
Downregulation of *KLF4* rescues miR-543's oncogenic effect on CRC cell proliferation, apoptosis, migration, and invasion in HCT116 cells **(A)** KLF4 protein expression was detected in HCT116 cells co-transfected with miR-543 inhibitor/miR-NC and *KLF4* siRNA or si-NC. **(B–E)** Cell proliferation, apoptosis, migration and invasion were assessed in HCT116 cells co-transfected with miR-543 inhibitor/miR-NC and *KLF4* siRNA or si-NC. * *p* < 0.05; the data represent the mean ± SD from triplicate measurements.

To investigate whether *KLF4* is required for the miR-543-mediated effects in CRC cells, HCT116 cells were co-transfected with the miR-543 inhibitor or miR-NC and *KLF4* siRNA. The transfection efficiency was assessed usign western blot analysis (Figure 6A). Our results revealed that *KLF4* downregulation promoted the miR-543-mediated oncogenic effects on cell proliferation, migration, apoptosis, migration and invasion in HCT116 cells (Figure 6B–6E). These findings further validated that miR-543 enhances cell proliferation, migration, and invasion, and inhibits apoptosis in CRC cells, at least in part by targeting *KLF4*.

## DISCUSSION

miRNAs have been reported to be involved in the progression and metastasis of human tumors. miRNAs plays a tumor suppressive or oncogenic role in human malignant tumors by targeting its downstream genes. As reported, miR-543 promotes gastric cancer cell proliferation by targeting SIRT1 [17]. However, Fan C et al reported that miR-543 affects cell proliferation and metastasis of CRC by targeting the expression of KRAS, MTA1 and HMGA2. Thus, miR-543 serves as a suppressor in CRC growth and metastasis [2]. Obviously, these results had conflicts. In spite of inconsistent results about miR-543 in the development of CRC, the exact mechanisms underlying miR-543-induced CRC proliferation, migration and invasion remained completely unclear.

In the present study, our results that miR-543 was overexpressed in human CRC tissues and cell lines. Also, miR-543 expression was obviously higher in these patients with TNM and larger tumor size. Spearman's correlation analysis also showed that the expression of miR-543 was positively correlated with tumor size. These findings revealed that miR-543 may play a crucial role in modulating cell proliferation and metastasis of CRC. Furthermore, the invasion and metastasis of CRC cells with miR-543 inhibitors was also affected *in vitro* and *in vivo*. These results indicated that miR-543 plays an important role in the development and progression of CRC.

It should be noted that epithelial-to-mesenchymal transition has been demonstrated as an important progression in cell invasion and metastasis of cancers. The role of miR-543 in EMT is less explored. In our study, downregulation of miR-543 in HCT116 and SW-480 cells exhibited an epithelial-shaped phenotype, increased E-cadherin expression and decreased vimentin expression. EMT-associated genes Twist, Snail, Slug and Zeb2 were also decreased due to knockdown of miR-543. These findings provided new evidence that miR-543 induces EMT of CRC cells. So, miR-543 is a crucial oncogene that enhances cell proliferation and metastasis in CRC.

To identify the mechanisms underlying miR-543-mediated effects in CRC, we further demonstrated Krüppel-like Factor-4 (*KLF4*) as a direct target of miR-543. As reported, *KLF4* is a member of the KLF family of zinc-finger transcription factor. *KLF4* has been reported to be highly expressed in the gastrointestinal tract epithelium, and is correlated with cell proliferation, growth regulation and cell differentiation [18, 19]. Previous reports showed that miR-103 induced the endothelial dysfunction and atherosclerosis by suppressing *KLF4* expression [20]. In addition, *KLF4* has been identified as anti-oncogene and oncogenes in various kinds of cancers [21–24]. In gastric cancer, the role of *KLF4* has been featured as a tumor suppressor and acts as a prognosis predictor for survival of patients. Overexpression of *KLF4* obviously affected cancer cell proliferation, invasion and metastasis [25–28]. This work demonstrated *KLF4* as a direct target of miR-543 in CRC using dual-luciferase reporter assay and western blotting. Moreover, an inverse correlation between miR-543 and *KLF4* expression in CRC was found. These findings validated that upregulation of miR-543 may induce CRC cell proliferation, migration and invasiveness by KLF4-induced signaling pathway.

In conclusion, this study demonstrated that miR-543 was highly expressed in CRC, and correlated with tumor size and lymph node metastasis. These findings demonstrated that miR-543 promoted cell proliferation, migration and invasion and inhibited cell apoptosis. In addition, *KLF4* was a direct target of miR-543. miR-543 functions as an oncogene by inhibiting its target gene *KLF4* in CRC. Therefore, miR-543 may serve as a promising target for the treatment of CRC patients.

## MATERIALS AND METHODS

### Ethical statement

All procedures performed in studies involving human participants were in accordance with the ethical standards of the institutional and/or national research committee and with the 1964 Helsinki declaration and its later amendments or comparable ethical standards.

### Tissue specimens

CRC cancer tissues (T) were obtained from 92 patients who underwent primary surgery of CRC between March 2015 and February 2016 at the Second Affiliated Hospital of Dalian Medical University. Adjacent non-tumor tissues (ANT) located at least 5 cm from tumors were selected randomly from 20 of these patients and used as controls. These 20 CRC tissues and matched ANT were used for western blot analysis. Tissue samples were snap-frozen in liquid nitrogen after resection until use. The basic clinical characteristics of these patients were documented in Table 1. Patients’ consent and approval from the Ethics Committee of the Second Affiliated Hospital of Dalian Medical University were obtained.

### Cell culture and transfection

Human CRC cancer cells lines HCT116, SW-480 and HT29 were obtained from the American Type Culture Collection (Manassas, VA, USA). Human intestinal epithelial cell lines FHC were obtained from the Second Affiliated Hospital of Dalian Medical University. All cell lines were cultured in RPMI 1640 medium (Hyclone, Logan, UT, USA) supplemented with 10% fetal bovine serum (FBS) (Gibco-BRL, Invitrogen, Paisley, UK). Cells were cultured at 37 °C in a humidified atmosphere containing 5% CO_2_. The miR-543 inhibitor and the corresponding negative control (miR-NC) were purchased from GenePharma (Shanghai, China). *KLF4* siRNA (si-*KLF4*) and the negative control (si-NC) were obtained from ambion (Austin, TX, USA). Transfection was performed using Lipofectamine 2000 Reagent (Invitrogen, Carlsbad, CA, USA) following the manufacturer's instructions.

### RNA extraction and real-time PCR (qRT-PCR)

Total RNA was extracted from tissue samples and cell lines using Trizol (Invitrogen) according to the manufacturer's protocol. SYBR^®^ Premix Ex Taq™ II kit (TaKaRa, Dalian, China) was used to quantify the expression levels of mature miR-543, EMT-associated genes and *KLF4* according to the protocol provided. Relative expression of miR-543 EMT-associated genes and *KLF4* was measured using the comparative cycle threshold (*C*_t_; 2^−ΔΔ*C*t^) method. All reactions were run in duplicate. All primers were listed as below:

E-cadherin:

Forward 5’-CGAGAGCTACACGTTCACGG-3’

Reverse 5’-GGGTGTCGAGGGAAAAATAGG-3’;

Vimentin:

Forward 5’-GACGCCATCAACACCGAGT-3’

Reverse 5’-CTTTGTCGTTGGTTAGCTGGT-3’;

Twist:

Forward 5’-GTCCGCAGTCTTACGAGGAG-3’

Reverse 5’-GCTTGAGGGTCTGAATCTTGCT-3’;

Snail:

Forward 5’-TCGGAAGCCTAACTACAGCGA-3’

Reverse 5’-AGATGAGCATTGGCAGCGAG-3’;

Slug:

Forward 5’-CAACGCCTCCAAAAAGCCAA-3’

Reverse 5’-ACTCACTCGCCCCAAAGATG-3’;

KLF4:

Forward 5’-TCGGACCACCTCGCCTTACA-3’

Reverse 5’-CTGGGCTCCTTCCCTCATCG-3’;

### Cell proliferation assay

Cell proliferation was determined using Cell Counting Kit-8 (CCK-8) solution (Dojindo, Gaithersburg, MD, USA) and the cell-light 5-ethynyl-20-deoxyuridine (EdU) Apollo Imaging Kit (Ribobio, Guangzhou, China) in accordance with the manufacturer's protocol. For CCK-8 assay, cells were seeded at a concentration of 4 × 10^3^ cells/well into three replicate wells on a 96-well plate and treated with 10 μL/well of CCK-8 solution during the last 4 h of culture daily for four consecutive days. The absorbance at 450 nm was measured with a microplate reader. The EdU assay was performed according to manufacturer's instructions. After EdU incubation, cells were treated with 1× Apollo solution and then stained with Hoechst 33342 (Ribobio, Guangzhou, China). The EdU positive cells was visualized under a fluorescence microscope (Olympus, Tokyo, Japan) and the positive percentage was defined as the proliferation rate. All reactions were performed in duplicate.

### Flow cytometry apoptosis assay

Cell apoptosis was measured by FITC Annexin V Apoptosis Detection Kit II (BD Bioscience, Woburn, MA, USA) following the manufacturer's protocol. Cells were collected at 48 h after transfection and stained with propidium iodide and Annexin V-FITC solution in the dark for 15 min. Stained cells were detected by FACS Caliber flow cytometer (BD Bioscience) for early and late apoptosis analysis. All data were obtained from three independent experiments.

### Cell migration and invasion assay

To assess the migratory and invasive potential of cells *in vitro*, the migration and invasion assays were performed using transwell chambers with 8 μm pore (Corning star, Lowell, MA, USA). Briefly, 1 × 10^5^ transfected cells were suspended in 200 μL serum-free medium and added to the upper chamber of transwell chambers. After incubation for 24 h in a humidified atmosphere containing 5% CO_2_ at 37 °C, these migrated cells that had stuck to the lower surface of the membrane were fixed in 4% paraformaldehyde and stained with 0.1% crystal violet for 15 min. The number of migrated cells was counted from five randomly selected fields at 200× magnification using a microscope. For invasion assay, the transwell chambers were coated with matrigel (BD Bioscience) and other procedure is the same as migration assay. Each experiment was performed in triplicate.

### Vector construction and luciferase reporter assay

The 3′-UTR sequence of *KLF4* or the mutant (Mut) was cloned into the downstream of the firefly luciferase gene in the pmirGLO plasmid (Promega, Madison, WI, USA). miR-543 inhibitor and miR-NC and the report plasmid pmirGLO-KLF4 Wt/Mut were co-transfected into cells (HCT116 and SW-480) using Lipofectamine 2000 (Invitrogen, Carlsbad, CA, USA). After 48 h of transfection, luciferase activities were detected by Dual-Luciferase Reporter System (Promega, Madison, WI, USA) and Renilla-luciferase activities were measured for normalization. All experiments were performed three times.

### Western blot analysis

At 48 h after transfection with miR-543 inhibitor or miR-NC, western blot analysis was done. Total protein was isolated from cells or tissues using RIPA buffer (Beyotime, Haimen, China) and was separated by SDS gels and then transferred to the nitrocellulose membranes. Immunoblotting was performed using the appropriate primary antibodies and the band was visualized using enhanced chemiluminescence reagents ECL (Pierce, Rockford, IL, USA). The following commercial antibodies were used in this study: KLF4 (1:1000, Proteintech, Wuhan, China), E-cadherin (1:1000, Santa Cruz, CA, USA), vimentin (1:1000, Santa Cruz) β-actin (1:1000; Cell Signaling, Danvers, MA, USA). Data were obtained from three independent experiments.

### Xenograft model experiment

All animal experiments were performed according to institutional and international animal regulations. Animal protocol was approved by the Institutional Animal Care and Use Committee of The Second Affiliated Hospital of Dalian Medical University. Male severe combined immunodeficiency (SCID) mice (four weeks old) were purchased from Beijing HFK Bioscience CO., Ltd. (Beijing, China). HCT116 cells were transfected with lentivirus miArrest™ miR-543 inhibitor (Genecopeia, Rockville, MD, USA). HCT116 cells were subcutaneously injected (1 × 10^6^ cells per mouse) and through tail vein into the mice (2 × 10^6^ cells per mouse), respectively. The growth of tumors was measured every seven days. All mice were euthenized after five weeks (subcutaneous xenograft group) or eight weeks (lung metastases xenograft group), and the tumor nodules and lungs of the mice were removed. Tumor sizes were measured using a caliper, and tumor volume was calculated according to the following equation: tumor volume (mm^3^) = length (mm) × width (mm)^2^/2. Histological analyses were used to detect distant metastasis in lungs in H&E sections. A portion of tumor tissues was used for analysis of RT-qPCR.

### Statistical analysis

All the data were presented as means ± standard deviation (SD). Statistical analyses were performed using Prism 5.01 ware (GraphPad Software, San Diego, CA, USA). Differences were determined with the analysis of variance (ANOVA), Student's *t*-test, or a χ-square test as appropriate. The correlation between miR-543 level and tumor size or *KLF4* level was analyzed by Spearman analysis. Survival curves were plotted by the Kaplan-Meier method and analyzed by the log-rank test. *p* < 0.05 was regarded as statistically significant in all cases.
